# Rapid functional recovery in a Golden Retriever puppy with swimmer puppy syndrome following early conservative management including loose-loop technique: a case report

**DOI:** 10.3389/fvets.2026.1907404

**Published:** 2026-09-04

**Authors:** Livia Kroker

**Affiliations:** Center for Canine Rehabilitation and Physiotherapy, Dresden, Germany

**Keywords:** canine physiotherapy, canine rehabilitation, early intervention, flat puppy syndrome, limb abduction, pectus excavatum, swimmer puppy syndrome, taping

## Abstract

Swimmer puppy syndrome (SPS) is a rare developmental condition of unknown etiology. The syndrome is characterized by a puppy’s inability to adduct the limbs, resulting in lateral limb abduction and an inability to stand or walk. In this case, an almost 4-week-old female Golden Retriever presented for early physiotherapeutic evaluation. Adduction tapes were applied to the forelimbs and hindlimbs. Additionally, the treatment area was covered with a non-slip rubber mat. Further physiotherapeutic interventions were implemented. Intensive care was discontinued after 10 days. At the 16-month follow-up, the dog showed no clinically apparent gait abnormalities and had made a good functional recovery. Early recognition and conservative management, including physiotherapeutic intervention with simple mechanical support through loose-loop taping and environmental modifications, can result in rapid functional recovery in puppies with swimmer puppy syndrome.

## Introduction

Swimmer puppy syndrome (SPS) is a rare developmental condition of unknown etiology (1). Only a few studies have addressed this disorder. The syndrome is characterized by a puppy’s inability to adduct the limbs, resulting in lateral limb abduction and an inability to stand or walk. These abilities are normally acquired by 2–3 weeks of age (2, 3). Affected puppies typically move by performing swimming or paddling motions with their limbs. The syndrome is often accompanied by a flat chest (2, 3). It can occur in various dog breeds of different sizes and appears to be more common in small litters (4, 5). SPS has also been reported in other animal species (6, 7). The diagnosis is usually made on clinical signs alone. Historically, the disease was often considered untreatable and frequently resulted in euthanasia. Studies have described various treatment approaches, including intensive physiotherapy, bandaging, external splinting, and hospitalization (2, 3, 8, 9). This case is of particular interest because the intervention was initiated very early and was followed by rapid functional improvement. In addition, a follow-up examination after 16 months revealed no clinically apparent gait abnormality and indicated good functional recovery. The aim of this report is to describe the rapid functional recovery of a puppy with swimmer puppy syndrome following early conservative management, including loose-loop technique and environmental modification.

## Case presentation

**Figure 1:**
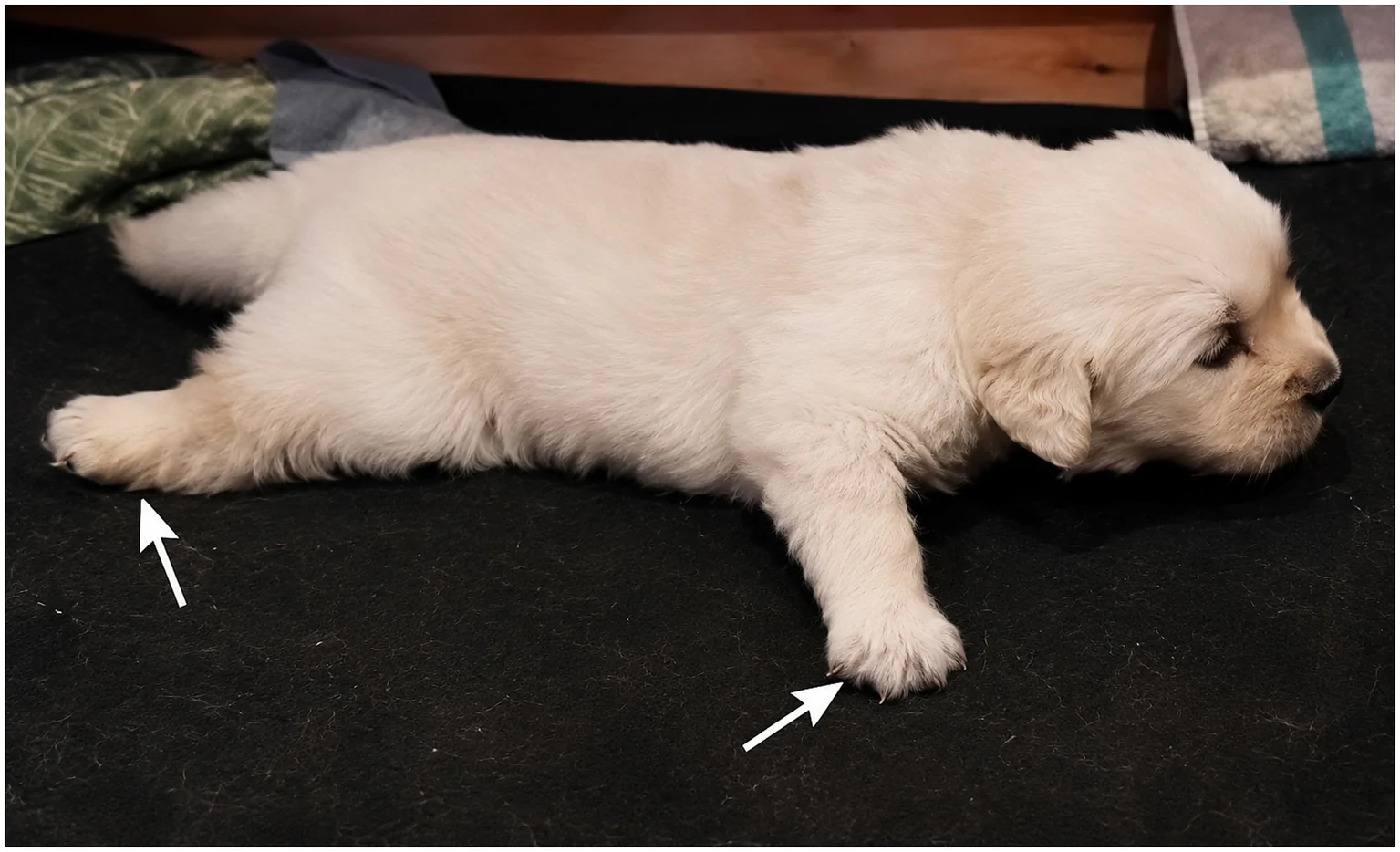
Golden Retriever puppy with swimmer puppy syndrome at approximately 4 weeks of age. The puppy shows the typical laterally abducted limb posture (white arrows) and an inability to stand, resulting in a swimming-like locomotion pattern.

A female Golden Retriever puppy, Charlotte, aged almost 4 weeks, was presented for physiotherapeutic evaluation. Unlike her littermates, the puppy had not yet developed the ability to stand or walk. The breeder described that, when she tried to stand, her hindlimbs would slip backward and her forelimbs would slip to the side. The puppy moved by performing characteristic swimming-like motions with her limbs (Figure 1). The litter consisted of only three puppies, and one male puppy died during birth.

**Figure 2:**
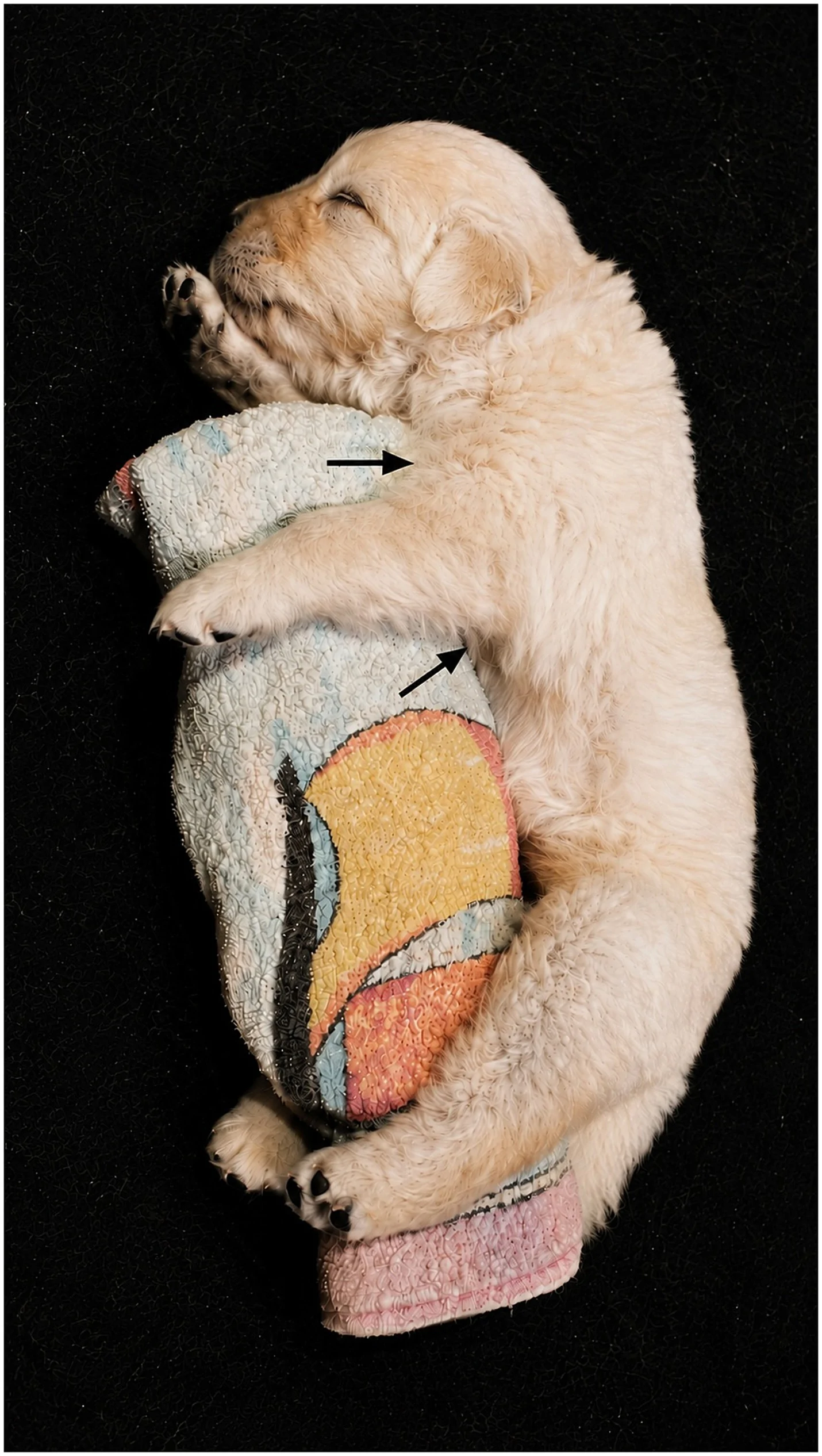
Flattened thoracic conformation (pectus excavatum) observed during early clinical presentation, indicated by the black arrows.

Charlotte had a reported birth weight of 460 g, which is within the normal range for Golden Retriever neonates. The surviving littermate had a birth weight of approximately 560 g. A flattened thoracic conformation consistent with pectus excavatum was already noticed by the breeder at approximately 1 week of age (Figure 2). In the weeks that followed, the puppy had difficulty crawling and reaching the dam’s mammary glands. As a result, her milk intake appeared reduced, and the puppy fatigued quickly during nursing. At approximately 4 weeks of age, when the puppy was first presented for physiotherapeutic evaluation, her body weight was 1,790 g. At that time, the littermate weighed approximately twice as much.

Despite the early growth delay, the puppy subsequently demonstrated rapid catch-up growth after receiving physiotherapeutic interventions and exhibited improved mobility. At 8 weeks of age, the body weight had increased to 5,650 g.

The puppy had previously been examined by two independent veterinarians, both of whom diagnosed her with swimmer puppy syndrome and referred her for physiotherapeutic treatment. Written records of the initial orthopedic and neurological examinations performed by these veterinarians were not available. Therefore, detailed findings from these examinations, including spinal reflex testing, were not available for analysis. Subsequently, focused orthopedic and neurological assessments were performed by a physiotherapist. The puppy was alert and active. The forelimbs and hindlimbs were held in an abducted position, and dorsoventral flattening of the thorax was evident. No swelling or obvious deformities of the limbs were observed. The passive range of motion of the joints was unrestricted, and no signs of pain were observed during the passive examination.

Differential diagnoses considered included congenital neuromuscular disorders, including disorders affecting the muscles, neuromuscular junctions, and peripheral nerves or motor neurons; orthopedic conditions, including angular limb deformities, congenital joint luxation, fractures, and malunion; neurological disorders, including inflammatory disease, brain or spinal cord malformations, trauma, and early-onset neurodegeneration; generalized neonatal weakness; severe nutritional compromise; and developmental delay (1). However, the characteristic limb abduction, a flattened thorax, unrestricted passive range of motion, the absence of signs of pain, and concordant previous assessments by two independent veterinarians supported the clinical diagnosis of swimmer puppy syndrome.

**Figure 3:**
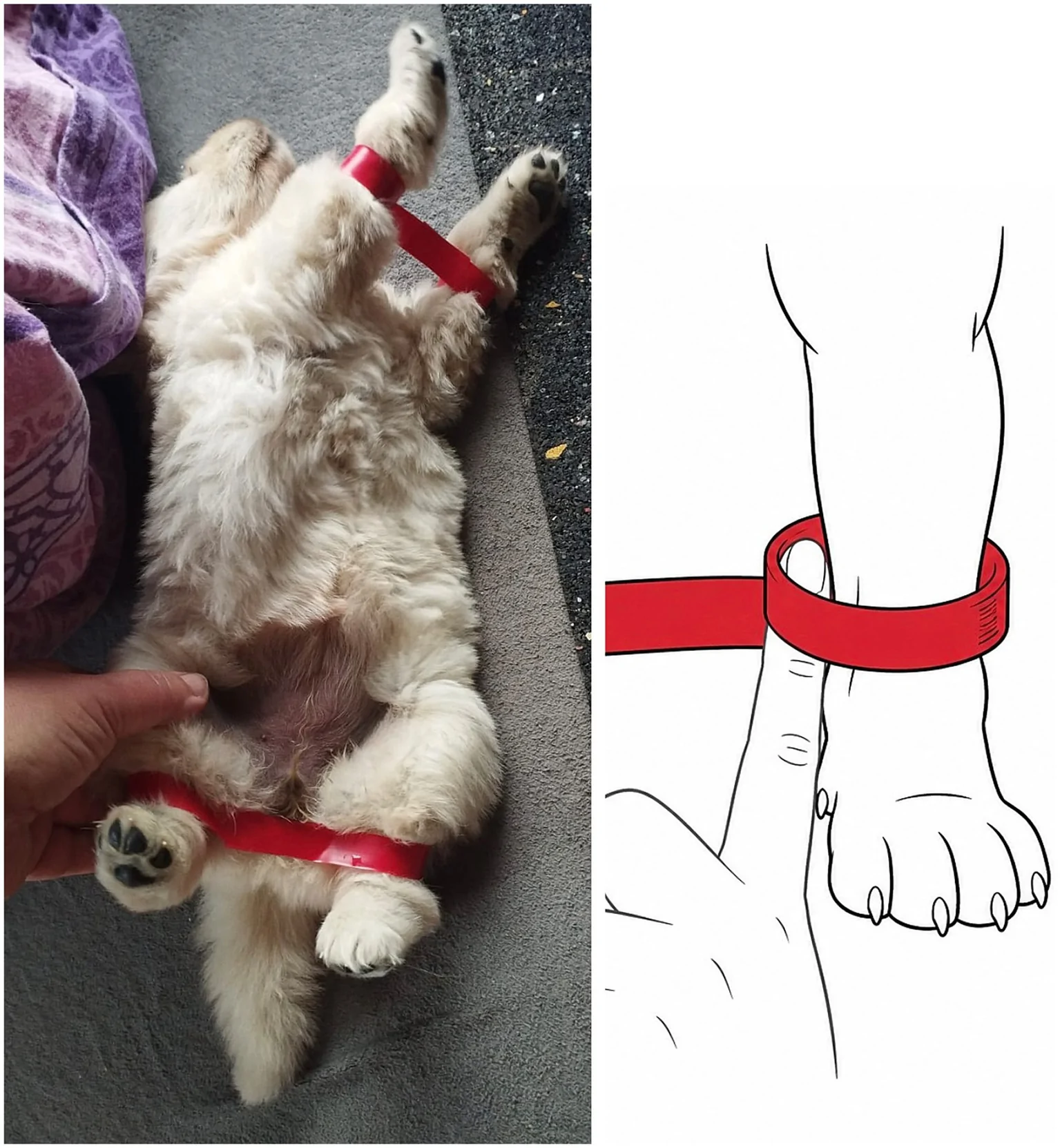
Representation of the “loose-loop technique.” The tape is applied around the distal limbs in a loose configuration, allowing approximately one finger space between the tape and the limb. The schematic illustration was generated using AI-assisted image generation and edited by the author.

During the first physiotherapy session, limb abduction was addressed using a simple adhesive taping configuration (commercial insulating tape) that allowed for the controlled restriction of lateral limb movement, while maintaining sufficient mobility to permit standing and stepping. A commercially available polyvinyl chloride (PVC) insulation tape (16 mm wide), purchased from a hardware store, was used. Tape dimensions may need to be adjusted to the patient’s size. Adduction tapes were applied to the forelimbs at the level of the antebrachial region and to the hindlimbs at the level of the crural region. The tape was manually shaped into a loose loop around the limb. During the application, the physiotherapist placed her little finger between the limb and the tape to ensure sufficient spacing. The two ends of the tape were then pressed together between the thumb and index finger, thereby securing the tape while maintaining the loose-loop configuration. In this configuration, the adhesive surface of the tape was in loose contact with the fur in some areas but did not touch the skin directly. For the purpose of description, this taping configuration may be referred to as the “loose-loop technique” (Figure 3). Importantly, the tape was not applied tightly to minimize the risk of skin irritation, edema, swelling, or circulatory compromise. During home management, the puppy remained under close supervision while the tape was in place. The breeder was instructed to regularly inspect the limbs for signs of swelling, skin irritation, or impaired function. For removal, the tape was cut with scissors and gently removed in the direction of hair growth. No adverse effects, including skin irritation, swelling, or edema, were observed during the treatment.

In addition, the treatment area was covered with a non-slip rubber mat. The breeder was instructed to continuously line the floor of the whelping box with non-slip mats to provide traction.

The treatment protocol for the first 3 days following the initial physiotherapy session was as follows: The tape was applied for approximately 3–4 h at a time and replaced three times daily, resulting in a near-continuous application throughout the day. It remained in place during both sleeping and active periods and was removed overnight. The passive joint mobilization of the forelimbs and hindlimbs was performed twice daily, in the morning and in the evening, for approximately 2–3 min per session. Each joint was gently moved through its available range of motion to the end ranges of flexion and extension. In addition, the back and abdominal regions were massaged using simple effleurage techniques (gentle stroking movements) for approximately 2–3 min twice daily. The sensory stimulation of the paws was performed using a soft toothbrush. Assisted standing exercises were performed two to three times daily for approximately 1–2 min per session. However, these standing exercises were considered of limited necessity, as the puppy had already learned to walk with the tapes in place. Additionally, supported water-treading exercises were performed once daily in a bathtub for approximately 1–2 min. A linen bag was modified by cutting four openings through which the puppy’s limbs were placed, thereby serving as a supportive sling. This bag was held manually to support the puppy’s body while allowing the limbs to move freely in water and perform active stepping movements.

Standing and walking abilities were already achieved during the first physiotherapy consultation (Table 1). Although the gait was unsteady on the first day, gait stability improved markedly by the second and third day. From the second day, the puppy was able to play with her littermate. The puppy actively participated in the exercises and showed no signs of stress, resistance, or fatigue during the treatment sessions. Lateral recumbency had already been performed by the breeder before the initial physiotherapy session and was continued thereafter. The puppy’s regular sleep periods were used for this purpose, with her position being corrected as needed. The breeder reported strong adherence to the prescribed home-management protocol, and no treatment-related adverse events were observed.

**Table 1:** Timeline of clinical findings, physiotherapeutic interventions, and functional recovery.

Age/time point	Findings/intervention
1 week	Thoracic flattening first observed by the breeder
3 weeks	Initial veterinary examination, SPS diagnosis
4 weeks	Physiotherapy assessment
Day 0	Loose-loop technique initiated and standing and walking achieved during the first treatment session
Day 3	Puppy climbed out of the whelping box overnight without tape support
Day 4	Independent walking without tape support
Day 10	Tape discontinued
8 weeks	Body weight: 5,650 g
16 months	No clinically apparent gait abnormality and good functional recovery

In addition, a vitamin B complex preparation and a trace supplement were administered orally for nutritional support.

On day 3 after the initiation of treatment, the puppy was able to leave the whelping box independently. By her second physiotherapy appointment, only 4 days after the first appointment, the dog was already walking without the tape. The tape was applied for a total of 10 days. Intensive care was discontinued after 10 days.

**Figure 4:**
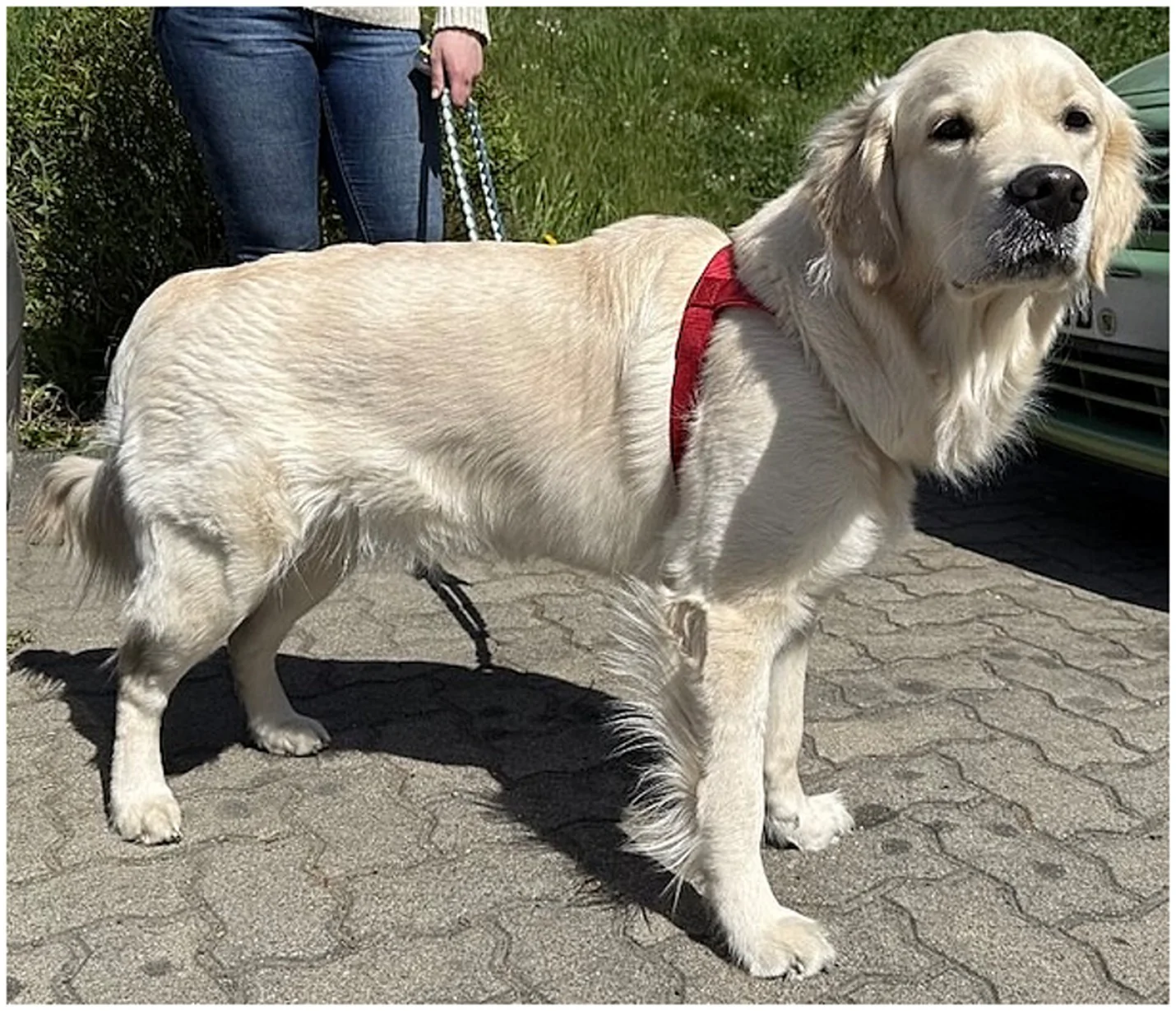
Long-term follow-up at 16 months of age demonstrating no clinically apparent gait abnormality, good functional recovery, and clinically normal thoracic conformation.

At a follow-up examination 16 months later, no clinically apparent gait abnormalities were observed, indicating good functional recovery (Figure 4). A follow-up veterinary examination revealed that the chest had fully expanded.

## Discussion

Swimmer puppy syndrome (SPS) is an uncommon developmental condition characterized by splayed limbs and an inability to stand or walk during the first weeks of life. The condition is often accompanied by thoracic flattening (pectus excavatum) and abnormal limb posture. Although the syndrome has been described for several decades in several dog breeds, its underlying pathophysiology remains poorly understood.

The largest epidemiological studies available to date suggest that SPS is associated with small litter sizes (1, 4, 5). The affected puppy in the present case originated from a litter of only two surviving puppies, consistent with these observations.

The pathophysiology of SPS remains unclear. Previous investigations have proposed several potential abnormalities, including neuromuscular immaturity, primary muscular abnormalities, genetic predisposition, and environmental or biomechanical influences (5). Similar hypotheses have been discussed in the analogous congenital splay leg syndrome in piglets (6, 10). Despite decades of research, no single causative mechanism has been conclusively demonstrated. Reports of entire affected litters have been described in kittens, further supporting the possibility of an underlying hereditary predisposition, although definitive genetic mechanisms remain unproven (7). Recent reviews suggest that the syndrome is likely multifactorial in origin and results from a complex interaction of developmental, genetic, and environmental factors (1, 5, 10).

The frequent occurrence of pectus excavatum in affected puppies has led authors to propose that thoracic flattening may develop secondary to prolonged sternal recumbency rather than representing a primary developmental defect (3). In the present case, marked thoracic flattening was evident during the early stages of the disease and was no longer apparent at follow-up after the development of normal mobility. This observation supports the proposed association. However, no causal relationship can be inferred because objective measurements or diagnostic imaging were not performed.

Consistent with previous reports, the majority of affected puppies achieve a complete recovery following appropriate interventions. Therefore, the condition is generally considered a reversible developmental disorder with a favorable prognosis (1–3, 8, 9).

Several therapeutic approaches have been described in the literature, including limb taping or bandaging, environmental modifications using non-slip surfaces, physiotherapy, positioning strategies, and supportive nursing care (2, 3, 8, 9).

Both the case report by Yardimci et al. (2) and the report by Kim et al. (3) described favorable outcomes following combinations of these interventions. Kim et al. reported a complete recovery of a miniature schnauzer puppy following a home-based management that included physiotherapy, limb support, and environmental modification, further supporting the effectiveness of early conservative treatment strategies in cases of SPS.

More recently, Rumpel et al. (1) reported that physiotherapeutic management was among the most commonly applied treatment strategies and emphasized the importance of early intervention. Karcher et al. (8) described favorable outcomes after using a modified bandaging technique based on immobilization. In contrast to these more restrictive bandaging approaches, the loose-loop technique was intended to support functional movement during early motor development. Rather than fixing the limbs in a predetermined position, the tape configuration provides gentle mechanical guidance while allowing the puppy to actively stand, step, and explore weight-bearing, requiring only minimal materials. The loose-loop technique represents an individual, non-validated mechanical support approach rather than an established therapeutic taping method. The safety of commercially available PVC insulation tapes in neonatal puppies has not been systematically evaluated.

The present case demonstrates rapid functional recovery following the implementation of a structured conservative treatment regimen consisting of physiotherapeutic intervention, environmental modifications, and a specifically designed loose-loop technique configuration. The tape was intentionally applied with minimal tension to preserve active movement while providing mechanical guidance during early motor development. At the 16-month follow-up, the dog in this study showed no clinically apparent gait abnormality and exhibited good functional recovery.

The rapid restoration of the ability to stand observed immediately following mechanical guidance is an interesting finding in the present case. This association may suggest that limb positioning and surface traction were relevant functional factors in this case. However, several therapeutic interventions were introduced concurrently. Therefore, no causal relationship between mechanical guidance and functional recovery can be established from this case. Although causality cannot be established from a single case, the clinical response observed in the present puppy should be interpreted as hypothesis-generating.

This report has several limitations. As a single case report, the findings cannot be generalized to all puppies affected by SPS. A further limitation is that detailed records of the original neurological examinations, including spinal reflexes, were unavailable. No objective imaging or additional diagnostic testing was performed. The diagnosis was therefore clinical.

Furthermore, multiple interventions were applied simultaneously, including environmental modification, nutritional support, and physiotherapy, making it impossible to determine the individual contribution of each treatment component. Vitamin B complex and trace-element supplementation were provided as supportive measures. Their individual contribution to recovery cannot be determined. Nevertheless, the detailed clinical documentation, photographic evidence, and long-term follow-up support the validity of the observations presented.

In conclusion, early multimodal conservative management, including physiotherapy and a simple loose-loop technique strategy, was associated with rapid functional recovery in this Golden Retriever with SPS.

The loose-loop technique is inexpensive, easy to apply, and designed to preserve active movement while providing gentle mechanical support during early motor development. Further studies are needed to evaluate the effectiveness and safety of this approach and to better understand the underlying mechanisms involved in SPS.

### Patient perspective

The breeder first noticed thoracic flattening at approximately 1 week of age. The characteristic signs of SPS became apparent later, at approximately 4 weeks of age, when the puppy was unable to stand or walk independently. According to the breeder, the puppy had difficulty crawling and reaching the mammary glands of the dam, resulting in reduced milk intake, slower weight gain, and increased fatigue during nursing. Following implementation of the physiotherapy program and loose-loop technique, improvement was observed immediately. The breeder described the treatment as easy to perform at home and was satisfied with the rapid recovery achieved.

## Data Availability

The original contributions presented in the study are included in the article/Supplementary material, further inquiries can be directed to the corresponding author.
